# Moxibustion for cancer care: a systematic review and meta-analysis

**DOI:** 10.1186/1471-2407-10-130

**Published:** 2010-04-07

**Authors:** Myeong Soo Lee, Tae-Young Choi, Ji-Eun Park, Song-Shil Lee, Edzard Ernst

**Affiliations:** 1 Division of Standard Research, Korea Institute of Oriental Medicine, Daejeon, South Korea; 2 Complementary Medicine, Peninsula Medical School, Universities of Exeter & Plymouth, Exeter, UK

## Abstract

**Background:**

Moxibustion is a traditional Chinese method that uses the heat generated by burning herbal preparations containing *Artemisia vulgaris *to stimulate acupuncture points. Considering moxibustion is closely related to acupuncture, it seems pertinent to evaluate the effectiveness of moxibustion as a treatment of symptoms of cancer. The objective of this review was to systematically assess the effectiveness of moxibustion for supportive cancer care.

**Methods:**

We searched the literature using 11 databases from their inceptions to February 2010, without language restrictions. We included randomised clinical trials (RCTs) in which moxibustion was employed as an adjuvant treatment for conventional medicine in patients with any type of cancer. The selection of studies, data extraction, and validations were performed independently by two reviewers.

**Results:**

Five RCTs compared the effects of moxibustion with conventional therapy. Four RCTs failed to show favourable effects of moxibustion for response rate compared with chemotherapy (n = 229, RR, 1.04, 95% CI 0.94 to 1.15, P = 0.43). Two RCTs assessed the occurrence of side effects of chemotherapy and showed favourable effects of moxibustion. A meta-analysis showed significant less frequency of nausea and vomiting from chemotherapy for moxibustion group (n = 80, RR, 0.38, 95% CIs 0.22 to 0.65, P = 0.0005, heterogeneity: χ^2 ^= 0.18, P = 0.67, I^2 ^= 0%).

**Conclusion:**

The evidence is limited to suggest moxibustion is an effective supportive cancer care in nausea and vomiting. However, all studies have a high risk of bias so effectively there is not enough evidence to draw any conclusion. Further research is required to investigate whether there are specific benefits of moxibustion for supportive cancer care.

## Background

Most cancer patients experience multiple symptoms related to either the cancer itself or late treatment effects [1]. The frequently experienced and severe adverse events associated with such treatments lead patients to seek supportive complementary and alternative medicine (CAM) [2]. Most patients use CAM as an adjunct to conventional treatments [3-5]. Acupuncture type interventions are one of the most popular forms of CAM [6]. It is now a widely accepted intervention for the treatment of a variety of conditions [7]. Several reviews claim that acupuncture offers therapeutic benefits for cancer patients [8-10]. Moxibustion is a traditional Chinese method that uses the heat generated by burning herbal preparations containing *Artemisia vulgaris *to stimulate acupuncture points [11]. There are two types of moxibustion. Direct moxibustion is applied directly to the skin surface at the acupuncture point [11]. In indirect moxibustion some insulating materials (ginger, salts and etc) were placed between the moxa cone and the skin [11]. Considering moxibustion is closely related to acupuncture, it seems pertinent to evaluate the effectiveness of moxibustion as a treatment of symptoms of cancer. Several reviews of moxibustion for cancer care are currently available [12-16]. However, most of these review failed to employ systematic and transparent methods and are open to bias [12,14-16]. Furthermore, they did not focus on moxbustion and do not provide specific evidence for moxibustion during cancer care. One overview [13], was not also comprehensive and open to selection bias. Currently, no systematic review of this subject is available. The aim of this systematic review was to critically evaluate all of currently available randomised clinical trials regarding the effectiveness of any type of moxibustion as adjunct therapy during cancer care.

## Methods

### Data sources

The following databases were searched from inception through to February 2010: MEDLINE, EMBASE, CINAHL, PychInfo, five Korean Medical Databases (Korean Studies Information, DBPIA, Korea Institute of Science and Technology Information, KoreaMed, and Research Information Center for Health Database), Chinese Medical Database (China National Knowledge Infracture: CNKI), The Cochrane Library 2010, Issue 1 and Japan Science and Technology Information Aggreator, Electronic (J-STAGE). The search terms were used as follows: (moxibustion OR moxa*) AND (cancer OR metasta$ OR carcinoma OR oncolo$ OR malignan$) in Korean, Chinese, or English. Reference lists of all obtained papers were searched in addition. We also performed electronic searches of relevant journals (FACT [Focus on Alternative and Complementary Therapies], and Research in Complementary Medicine [Forschende Komplementarmedizin] up to January 2010) through their website. Further, our own personal files were manually searched. Hardcopies of all articles were obtained and read in full.

### Study selection

Prospective randomised clinical trials (RCTs) were included if moxibustion was used as the sole treatment or as an adjunct to other treatments for patients having any type of cancer (if the control group also received the same concomitant treatments as the moxibustion group) and if clinically relevant outcomes were assessed. Trials with designs that did not allow an evaluation of efficacy of the test intervention (eg, by using a treatments of unproven efficacy in the control group or comparing two different forms of moxibustion) were excluded. Trials were also excluded if only immunological or biological parameters were accessed. Trials published in the forms of dissertation and abstract were included. No language restrictions were imposed.

### Data extraction and assessment of risk of bias

Hard copies of all articles were obtained and read in full. All articles were read by three independent reviewers (MSL, TYC, SSL) and data from the articles were validated and extracted according to pre-defined criteria (Table 1). No language limitations were imposed.

**Table 1:** Summary of parallel open, randomised clinical studies of moxibustion for cancer

First author (year)	Sample size Condition	Intervention group (Regimen)	Control group (Regimen)	Main outcomes	Intergroup differences	Treated acupuncture points Rationale for point selection
Cheng (2005) [18]	84 Nasopharyngeal carcinoma	(A) Moxibustion (once daily for 30 days, n = 42), plus radiotherapy and chemotherapy Indirect	(B) Chemotherapy and radiotherapy, plus drug therapies for side effects (n = 42)	Response rate	NS, 1.05 [0.95, 1.16]	CV8 n.r.
Chen (2000) [19]	56 Nasopharyngeal carcinoma	(A) Moxibustion (once daily for 30 days, n = 28), plus (B) Indirect	(B) Chemotherapy and radiotherapy (n = 28), plus drug therapies for side effects	1) Response rate 2) Side effect of chemotherapy3) 5-year survival rate	1) NS, 1.10 [0.83, 1.44] 2) P < 0.05 in favour of A 3) NS, 1.40 [0.75, 2.60]	CV8 n.r.
Cao (1997) [20]	36 Gastric cancer	(A) Moxibustion (3 times weekly, n.r, n = 12), plus (B) Indirect	(B) Chemotherapy(n = 12) *(C) Chemotherapy plus drug therapies for side effects (n = 12)*	1) Response rate 2) Side effect of chemotherapy	1) NS, 2.0 [0.82, 2.34] 2) P < 0.05 in favour of A	CV8 n.r.
Liu (2001) [21]	81 Various cancer (Malignant tumor)	(A) Moxibustion (once daily, n.r, n = 30), plus (B) Indirect	(B) Chemotherapy (n = 35), plus herbal medicine (Gubenyiliu III 400 ml, twice a day) *(C) Chemotherapy (n = 16)*	1) Response rate 2) Living quality	1) NS, 0.99 [0.87, 1.12] 2) NS, 0.22 [-0.27, 0.71]	GV14, BL17, ST36 n.r.
Bian (2004) [22]	44 Various cancer (cancer pain)	(A) Moxibustion (2-3 times daily for 20 days, n = 23), plus morphine injection (acupoint, 5-10 mg, twice a day) Indirect	(B) Morphine injection (10-20 mg, 2-3 times a day, n = 21)	Living quality	P < 0.00001, 2.03 [1.29, 2.78] in favour of A	GV14, CV4, ST36, LI4, ashi-point n.r.

NS: not significant; n.r.: not reported

Risk of bias was assessed using the Cochrane classification in four criteria: sequence generation, blinding, incomplete outcome measures, and allocation concealment [17]. Considering that it is hard to blind therapists to the use of moxibustion, we assessed patient and assessor blind separately. If it is patient-assessed pain then it is not possible to assessor blind because the patient himself would be the assessor. The assessor needs to be a different person. Thus, if pain is assessed by another person (not the patient himself) then assessor blinding would be possible. Disagreements were resolved by discussion between the two reviewers (MSL, TYC). There were no disagreements between the three reviews about the risk of bias.

### Data synthesis

To summarise the effects of moxibustion on outcomes (response rate), we abstracted the risk estimates (relative risk: RR) and and 95% confidence interval (CI) was calculated using the Cochrane Collaboration's software (Review Manager (RevMan) Version 5.0 for Windows. Copenhagen: The Nordic Cochrane Centre). For studies with insufficient information, we contacted the primary authors to acquire and verify data where possible. If appropriate, we then pooled the data across studies using random effects models (if excessive statistical heterogeneity did not exist). The chi-square test, and the Higgins I^2 ^test were used to assess heterogeneity.

## Results

### Study description

**Figure 1:**
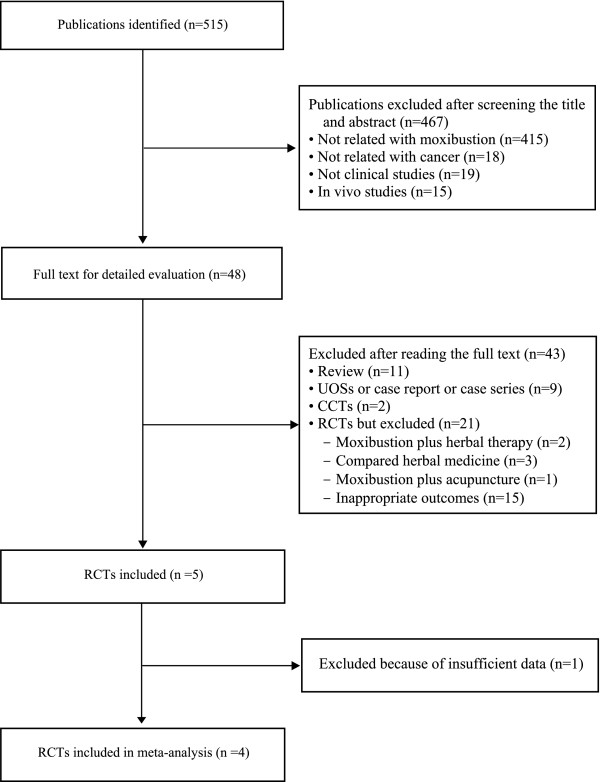
**Flowchart of trial selection process**. RCT: randomized clinical trial; CCT: controlled clinical trial; UOSs: uncontrolled observational study

The searches identified 515 potentially relevant articles of which 510 studies were excluded (Figure 1). Key data of the included 5 RCTs are summarized in Table 1[18-22]. All trials originated from China. Three [18,19,22] of the included trials had a two-armed, parallel group design and two RCTs [20,21] used a 3-armed parallel group design. The types of cancer treated within the trials were gastric cancer [20], nasopharyngeal carcinoma [18,19], and various cancers [21,22]. The objective outcome measures were survival rate [19], response rate [18-21], and side effects of chemotherapy [19,20], and quality of life [21,22]. None of the included RCTs reported the rationale for selecting treatment points. All RCTs employed indirect moxibustion.

### Risk of bias

The most of included trials had high risk of bias. One RCT [19] employed appropriate sequence generation. None described incomplete outcome measures One study reported details about allocation concealment [19]. None assessed the adverse events from moxibustion.

### Outcomes

#### Response rate

**Figure 2:**
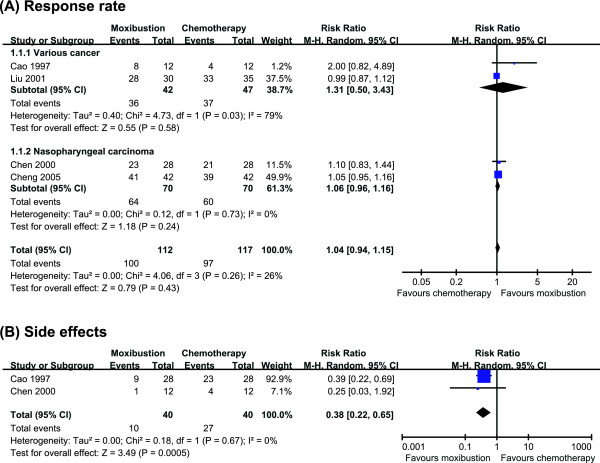
**A forest plot of moxibustion for cancer care**. (A) treating cancer, showing the response rate for moxibustion plus chemotherapy vs. chemotherapy; (B) side effects.

Four RCTs reported response rate for moxibustion as an adjunctive of chemotherapy compared with chemotherapy [18-21]. All of 4 RCTs failed to show favourable effects of moxibustion on response rate. The meta-analysis also suggested not significant difference between two groups (n = 229, RR, 1.04, 95% CI 0.94 to 1.15, P = 0.43, heterogeneity: χ^2 ^= 4.06, P = 0.26, I^2 ^= 26%, Figure 2A). Subanalysis also failed to show favourable effects of moxibustion on response rate in patients with nasopharyngeal carcinoma (n = 140, RR, 1.06, 95% CIs 0.96 to 1.16, P = 0.24, heterogeneity: χ^2 ^= 0.12, P = 0.73, I^2 ^= 0%, Figure 2A) [18,19].

#### Side effect of chemotherapy

Two RCTs assessed the occurrence of side effects of chemotherapy [19,20]. Both studies showed favourable effects of moxibustion plus chemotherapy compared with chemotherapy. A meta-analysis showed significant less frequency of nausea and vomiting from chemotherapy for moxibustion group (n = 80, RR, 0.38, 95% CIs 0.22 to 0.65, P = 0.0005, heterogeneity: χ^2 ^= 0.18, P = 0.67, I^2 ^= 0%, Figure 2B).

#### Quality of life

Two RCTs tested the effects moxibustion on quality of life compared with chemotherapy or morphine injection [21,22]. One RCT [22] showed favourable effects of moxibustion compared with morphine injection, while other RCT [21] failed to generate positive effects compared with chemotherapy.

## Discussion

This systematic review identified only very few RCTs for moxibustion. Their results fail to provide convincing evidence for the effectiveness of moxibustion. However, two RCTs demonstrate that moxibution as an adjunctive therapy is more effective for reduction of side effects (from chemotherapy) than chemotherapy alone [19,20], specifically for nausea and vomiting. In the present set of studies, an absence of adequate statistical analysis of the variability of therapeutic protocols and poor quality of reporting are frequent methodological problems. Collectively, the current evidence from RCTs of moxibustion as supportive cancer care is not convincing. However, the number of trials and the total sample size and their methodological quality are too low to draw firm conclusions.

The risk of bias in the studies was assessed based on the descriptions of sequence generation, blinding, incomplete outcome data, and allocation concealment. All of the studies were burdened with a high risk of bias. One RCT [19] employed allocation concealment and none of the RCTs made an attempt to blind assessors. One RCT [19] reported details of drop-outs and withdrawals, while the others didn't describe that may have led to exclusion or attrition biases. Thus the reliability of the evidence presented is clearly limited.

All of included trials compared indirect moxibustion with chemotherapy or morphine. The fact that there is no good trial evidence in support of moxibustion is in line with several different interpretations. Moxibustion may be ineffective, the studies may have been incorrectly designed or the treatment may not have been administered optimally in the existing studies. In the absence of a sufficient number of RCTs, other types of evidence might be helpful. Two controlled trials reported positive effects of moxibustion compared with chemotherapy, drug or no treatment in cancer patients [23,24]. Uncontrolled trials also imply that moxibustion is beneficial for symptom management of various cancers [25-29]. Unfortunately, such data are highly susceptible to bias and hence, they provide little useful information on the specific effects of moxibustion as it relates to supportive cancer care.

One argument for using moxubustion for the supportive care for cancer might be that it is safer than drug treatment. None of included trials assessed adverse events. Currently 3 studies evaluated the adverse events or possible risks of moxibution [30-32]. Mild or no adverse effects of were noted in previous reports [30,32], while one study [31] concerned possible hazardous in health by smoke from mouldering moxibustion. Relative to those of other conventional treatments, these are mild, infrequent and perhaps even negligible. Further study is needed to clarify this.

Assuming that moxibustuon is beneficial for cancer patients, possible mechanisms of action are of interest. Moxibustion may exert not only absorption of extract from moxa on acupuncture points but also direct effects due to acupuncture point stimulation from heat. Some aspect of mechanism may be similar that of acupuncture. One of them is that moxibustion may influence the multiple cortical, subcortical/limbic, and brainstem areas [33-37]. Involving these modulation therapeutic effects of moxibustion may mediate partially through opioidergic and/or monoaminergic neurotransmission [35,38]. Acupuncture often evokes complex somatosensory sensations and may modulate the cognitive/affective perception of pain, suggesting that many effects are supported by the brain and extending central nervous system networks [36,39,40]. Another possible mechanism includes an influence on the heat shock proteins and the function of immune cells. It has been shown that moxibustion up-regulated heat shock protein 70 and decreased the gastric injury and apoptosis of gastric mucosal cells [41]. The third hypothesis is that the moxibustion improves the function of immune cells. Moxibustion induced higher cellular immune function and increased the content of β-endorphin in the lymphocyte of the spleen in HAC cancer mice [42]. Moxibustion may modulate immunity through neurohormonal regulatory mechanism. Moxibustion also inhibited the growth of tumor and enhanced cellular immune functions via cytokine production (IL-2 or IL-12) [43] and increase of natural killer cell activity in tumor-bearing mice [44]. None of these theories are, however, currently fully established.

One could also argue about the value of conducting systematic reviews or meta-analyses of a limited number of included studies. They can increase power, improve precision, answer questions not asked by individual studies, settle controversies arising from conflicting results, improve the quality of future primary studies, and generate new hypotheses [45-47]. Systematic review can also avoid biases and make results and conclusions as objective as possible [46]. Even systematic reviews that find no primary studies to include can be valuable in that they may point towards important gaps in our knowledge. However, systematic reviews are retrospective and strongly depend on the quality of the primary studies [46]. They may also lead to contradictory overall conclusions [46]. The use of statistics does not guarantee that the results are valid. In our case, as the conclusions from the meta-analyses are from only 4 RCTs, the conclusions must remain tentative.

Limitations of our systematic review pertain to the potential incompleteness of the evidence reviewed. We aimed to identify all studies on the topic. The distorting effects of publication bias and location bias on systematic reviews are well documented [48-50]. In the present review there were no restrictions on the review publication language, and a large number of different databases were searched. We are therefore confident that our search strategy located all relevant data on the subject. Most of the included RCTs that reported positive results come from China, a country which has been shown to produce no negative results [51]. Further limitations include the paucity and the often suboptimal quality of the primary data.

## Conclusion

The evidence is limited to suggest moxibustion is an effective supportive cancer care in nausea and vomiting. However, all studies have a high risk of bias so effectively there is not enough evidence to draw any conclusion. Further research is required to investigate whether there are specific benefits of moxibustion for supportive cancer care.

## Competing interests

The authors declare that they have no competing interests.

## Authors' contributions

MSL conceived the study design. TYL, and JEP searched and selected the trials, extracted, analyzed and interpreted the data. MSL drafted the manuscript. TYC and SSL searched Chinese Databases and extract data from Chinese literatures. TYC updated the search and the content of the review. EE helped with the study design and critically reviewed the manuscript. All authors read and approved the final version of the manuscript.

## Pre-publication history

The pre-publication history for this paper can be accessed here:

http://www.biomedcentral.com/1471-2407/10/130/prepub
